# The Influence of Polystyrene and Biodegradable Microplastics on *Phaseolus vulgaris L.* Growth

**DOI:** 10.1021/acsomega.5c11468

**Published:** 2025-12-18

**Authors:** Tatiane Zucchini, Marcos Henrique Rodrigues Oliveira, Olavo Nardy, Eliane Trovatti

**Affiliations:** Department of Health and Biological Sciences, University of Araraquara (UNIARA), Rua Carlos Gomes, 1217, 14800-340 São Paulo, Brazil

## Abstract

The presence of microplastics (MPs) in the soil represents a growing threat to food security, affecting plant growth and ecosystem health. In this context, it is essential to investigate the effects of soils contaminated with different types of MPs on plant growth. This study evaluated, for the first time, the influence of two types of MPs (synthetic and biodegradable) on the growth of *Phaseolus vulgaris* L. Three types of soil were used: control (without MPs), soil loaded with biodegradable MPs, and soil loaded with expanded polystyrene MPs. Twelve seeds were individually buried in each soil sample. The lengths of the leaves and stems were measured on days 7, 14, 21, and 38 after planting. Fresh and dry biomass of leaves, stems, and roots were quantified, as well as the soil pH. The chlorophyll content was analyzed on days 7 and 38 by spectrophotometry. Microbiological analysis was performed at the beginning and at the end of the experiment to estimate the number of bacteria and fungi. The results indicated that soil with PS-MPs reduced the biomass of leaves and roots, showed irregular behavior in chlorophyll production, and lower microbial number, evidencing its negative effects on plant growth. The soil loaded with Bio-MPs did not show relevant changes when compared to the control soil. In general, the results indicated that PS-MPs negatively impact bean development, affecting its morphology and microbiota, leading the plant to stress and senescence.

## Graphical abstract

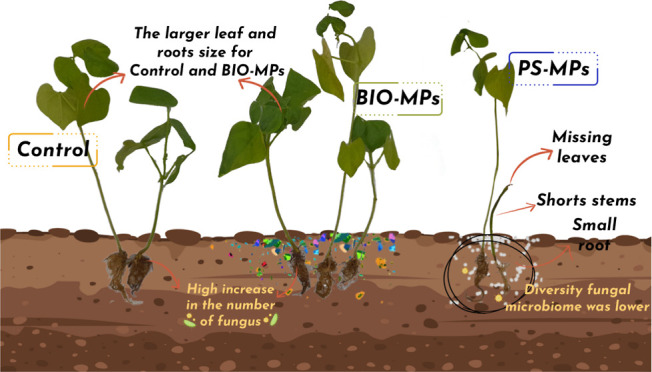

## 1 Introduction

The plastic journey started in 1862 with the “Parkensise” when Alexander Parkes introduced his innovation—the cellulose derivative molded by heating.^1^ In 1909, Leo Baekeland introduced the “bakelite”, the first completely synthetic plastic material, the phenol-formaldehyde.^2^ Properties such as thermal moldability, low density, mechanical strength, durability, water impermeability, transparency, and others have led plastics to be used in a large range of industrial applications and in daily routines. Many of these long-life and mechanically robust materials show slow degradation kinetics. The degradation process generates plastic microparticles, known as microplastics (MPs), which disperse and migrate throughout the environment and can be found almost everywhere. Common routes for the initiation of MP migration include, for instance, those produced in urban areas that accumulate on residential roofs and are carried by wind and rain into the soil.^3^ The plastic waste improperly disposed in landfills also represents a starting point of the migration route because after plastics disintegration and generation of small particles, they are leached with the water and spread in nature.^4^ The diagram of the possible plastic migration routes is shown in Figure 1.

**Figure 1:**
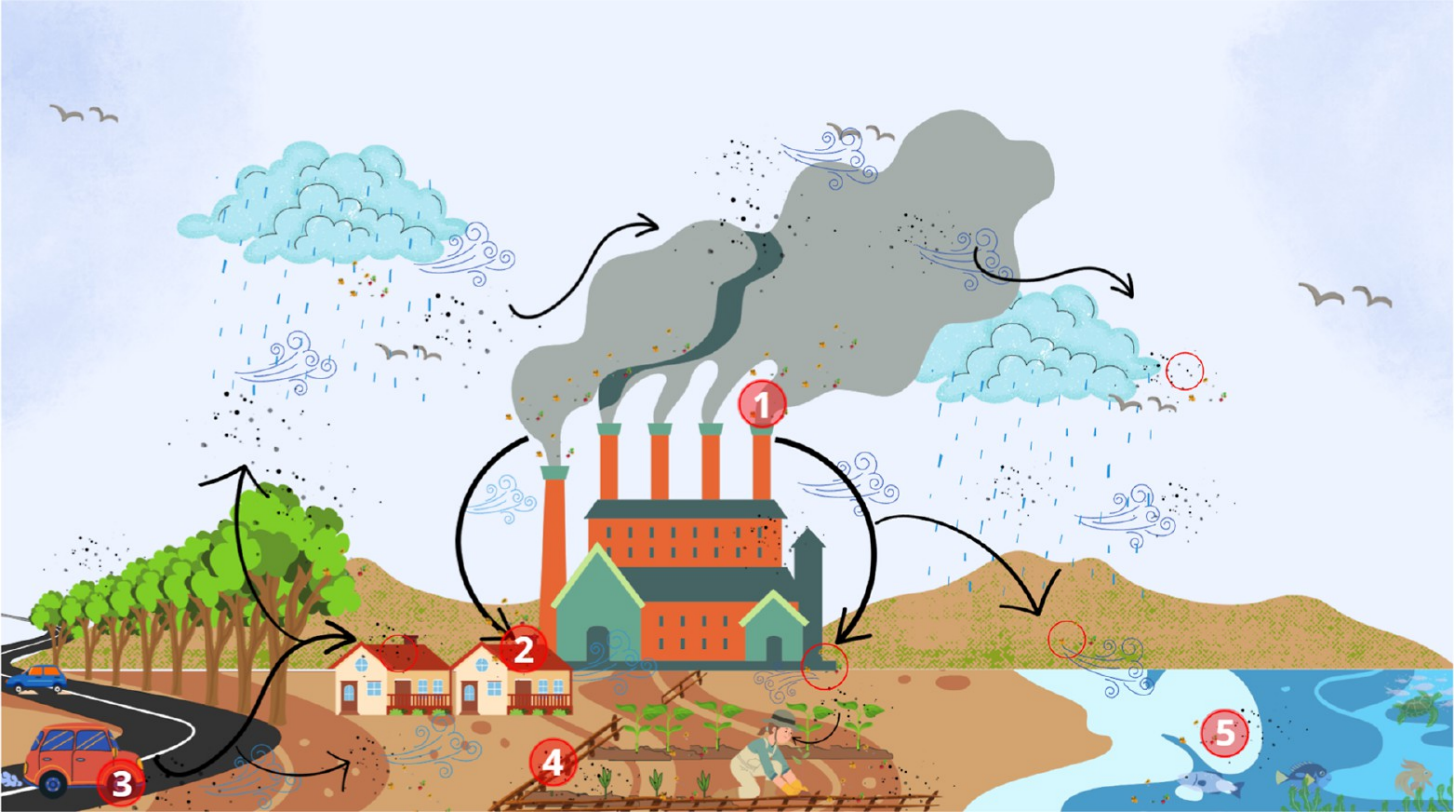
Representation of MP routes across terrestrial, food, and aquatic systems. Emissions from industrial chimneys (1), tire wear from vehicles (3), and wind transport contribute to the dispersion of MPs to urban areas (2), agricultural fields (4), and marine environments (5).

The abundance of MPs in agricultural soils has been analyzed in several countries, and the results show that the concentration and the polymers that give rise to the MPs are highly variable. For instance, approximately 0.12 items·kg^–1^ of polycarbonate (PC), acrylonitrile-butadiene-styrene (ABS), polypropylene (PP), low-density polyethylene (LDPE), high-density polyethylene (HDPE), polyethylene terephthalate (PET), ethylene-vinyl acetate (EVA), polystyrene (PS), and poly­(methyl methacrylate) (PMMA) mixtures can be found in Bangladesh, in rural farmlands, approximately 41.7 items·kg^–1^ of PP, polyethylene (PE), LDPE, polyamide (PA), PS, and others mixture can be found in vegetable soils in China, and approximately 541 items·kg^–1^ of PP, PE, PS, and acrylates mixture can be found in agricultural soils, in Canada.^5^ In general, the long-term effects of MPs include changes in soil pH, changes in the amount of available nitrogen, and changes in soil aggregation. All these parameters may either decrease or increase depending on the type of MPs, soil type, and time. Few positive effects of MPs in soils were also described, such as the stimulation of the nitrogen fixation and nitrogen degradation, resulting from the presence of PE and biodegradable MPs.^5^

In a general context, terrestrial ecosystems contaminated with MPs can compromise the interactions among soil microbiota, plants, and the environment, thereby influencing plant growth and leading to negative impacts on food production. One major concern of soil contamination by MPs is their potential effect on agricultural areas. Although it does not yet affect large-scale production, it should be considered in the near future since microplastic accumulation has been detected in the past few years, in soil. Agriculture covers about one-third of the land surface (suitable for cultivation) on the planet; however, food insecurity persists, leading to the concerns with the negative impact of MPs on food security to grow. Thus, the agricultural practices would need to be monitored aiming for sustainable food production, avoiding soil degradation, including the effects of MPs.^6^

The concerns regarding the influence of soil degradation in food security encouraged studies on this research field, which has been rapidly increasing. Several published studies show the influence of MPs on specific crops. For instance, large MP particles may adsorb at the surface of crop vegetables, and the smaller particles can be internalized into the vegetable system. These MPs can be found at high concentrations in fruits (apple and pear), vegetables (lettuce, cabbage, carrots, potatoes, and wheat), and cereals (wheat).^7^ Another study showed the microplastic accumulation in the leaves of watercress after 72 h of exposure in laboratory tests, and its association with the delay on the vegetable growth.^8^ The effect of microplastic on soil aggregation, water retention capability, and density, affecting, therefore, the soil aeration and plant root growth is also described.^9–12^ Studies carried out with spring onions and perennial ryegrass revealed changes in total plant biomass, as well as in seed germination rates, when soil contaminated with MPs were used for cultivation.^11,13^ Despite the growing number of publications in this field, several gaps remain regarding the influence of specific MPs on plant growth. To the best of our knowledge, no information is available comparing the effects of soil contamination with polystyrene MPs (PS-MPs) and biodegradable MPs (BIO-MPs) on *Phaseolus vulgaris* crops. Polystyrene is widely used in packaging, and biodegradable-based packages have been introduced to the market as alternatives to conventional packaging materials, thus warranting comparative studies on its derivative MPs influence on the plant growth when they contaminate soils.

*P. vulgaris* is useful as a model vegetable for studies of exposure to MPs because it is sensitive to soil conditions.^14–16^ *P. vulgaris* L. has already been grown in soil contaminated by MPs, such as the mixture of biodegradable plastics (PLA and PBAT). The dose-dependent results showed that at concentrations higher than 1.5% wt, the shoots and roots biomass decreased, and at doses higher than 2.0% wt, the leaf area and fruit biomass decreased.^17^ Divergent results were also described in the literature, for instance, for soil contaminated with small plastic beads (5 mm), microglitter, and broken-up Styrofoam, which did not show difference on the shoot growth, bean production, and total bean weight when compared to the noncontaminated soil.^18^

In this scenario, the main aim of this study was to determine the influence of MPs, namely, polystyrene and the compostable polymer, on *P. vulgaris* L. growth. For such, the vegetable was cropped in soil loaded with the MPs and its growth was followed by measuring the seedling incidence, plant morphology, quantifying the fresh and dry biomass of leaves, stems, and roots, and the length of leaves and stems. In addition, the pH of the soil was measured before crop and after sampling. Chlorophyll production and the number of bacteria and fungi were determined at the beginning and at the end of the experiments.

## 2 Materials

Soil (Green Garden, Brazil), expanded polystyrene ABNT NBR 11949 NBR11949, biodegradable plastic (sacco biodegrabile e compostabile conforme alla norma UNI EN 134:2002), *P. vulgaris* L. beans (Camil Carioca Tipo 1, Brazil), nutrient agar (Dinâmica, Brazil, CAS: 9002–18–0), nutrient broth (Kasvi, Italy, REF: K25–610,037), and Sabouraud dextrose broth EP-USP (Kasvi–Spain, CAS: K25–1205) were used.

## 3 Methods

*P. vulgaris* L. behavior was evaluated after growth in three soil conditions: healthy soil (control), soil with biodegradable plastic, and soil with expanded polystyrene, named as control, Bio-MPs and PS-MPs, respectively. The sample size was set at 12, with three replicates. Three replicates were removed from the soil and analyzed after 7, 14, 21, and 38 days. The samples were randomly removed for analyses.

### 3.1 Microplastic and Soil Preparation

The polymer was milled using conventional sandpaper and incorporated into the soil. The soil (150 mL, or 160 g) and the microplastic (30 mL) were mixed manually and used to fill the seedbed, using 15 mL of soil for each individual container. Such soil volume (30 mL) corresponds to 3.5 g for Bio-MPs and 0.6 g for PS-MPs. The control group is the soil free of microplastic. The concentration of MPs was based on the literature.^5^ Scanning electron microscopy (SEM) was performed to determine the particle size using a Jeol- JSM-6610LV scanning electron microscope operating at 12 kV. The air-dried microsplastic was deposited on the surface of an aluminum support, dried, and coated with evaporated platinum.

### 3.2 Bean Growth

Three seedbeds (11.5 cm × 9 cm) with 12 individual containers (15 mL) were used to grow the beans and labeled as control, polystyrene, and biodegradable. The individual containers were filled with 15 mL of each soil sample, and only one bean was buried approximately 1.5 cm from the surface. Thus, 12 seeds were grown individually. Watering was carried out every day, using 2 mL of water for each individual container. The bean incidence was visually analyzed every day, and each one was counted individually as one incidence unit/day. The scheme of the methodology is shown in Figure 2. During the study, the local temperature ranged from 17° to 34.9° degrees, 10 h of natural light incidence/day, according to meteorological measurements available at INMET (2023).^19^ The research was carried out in Brazil, at São Paulo state, Araraquara city, located at 21°47′40″ south latitude and 48°10′32″ west longitude. The experiment was followed, and pictures were acquired every day to evaluate the bean incidence and growth.

**Figure 2:**
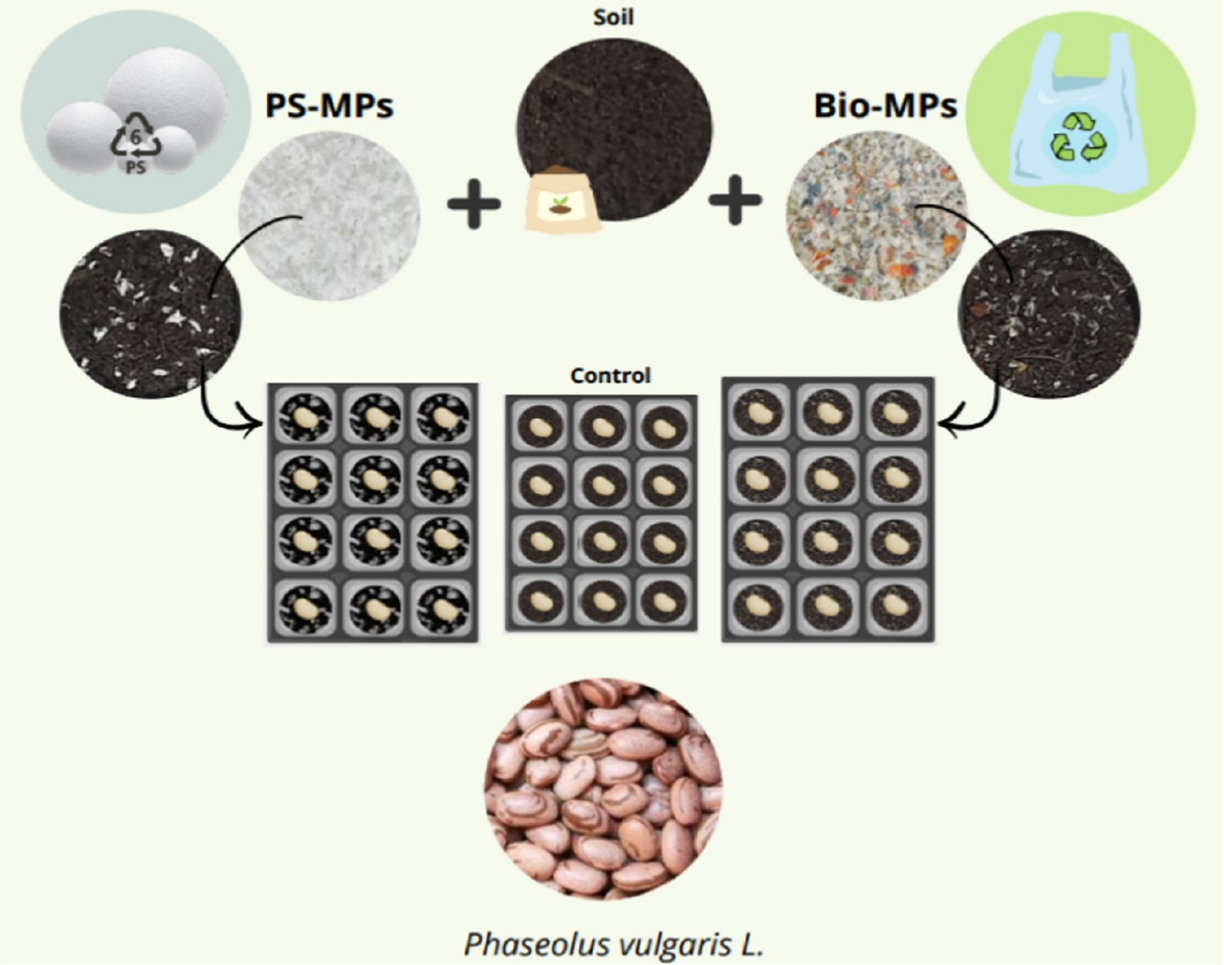
Schematic representation of the soil preparation with microplastic and *Phaseolus vulgaris* *L.* beans, displayed on the surface of the soil. Soil, PS-MPs, BIO-MPs, and beans are actual pictures, and the containers with 12 seeds are schematic representation.

### 3.3 Morphological Analysis

The morphological analysis was carried out at 7, 14, 21, and 38 days time point. Three samples were analyzed for each period of time. For such cases, the leaf length right and left, and stem length were measured using the pachymeter (Mitutoyo).

### 3.4 Fresh and Dry Biomass Determination

The seedlings were cut into three parts, leaves, stems, and roots, and weighed using an analytical balance to determine the fresh biomass. After the fresh biomass was measured, the biomass of the leaves and stem were dried in an oven at 50 °C until the mass kept constant. The experiment was carried out in triplicate.

### 3.5 Soil pH

The pH was measured at 7, 14, 21, and 38 days. The plant was removed from the containers, the soil was inserted into the beaker cup, and 100 mL of distilled water was poured into the beaker, homogenized for 1 min using a magnetic stirrer, and rest for soil sedimentation. Once the sedimentation was completed, water was used to determine the pH analysis using a Kasvi benchtop pH meter.

### 3.6 Chlorophyll Analysis

The chlorophyll absorbance spectrum was acquired using the UV–vis spectrophotometer BEL Engineering-VM5, for the samples harvested at 7 and 38 day time point. For such cases, the leaves were weighed (0.25 g) and gently homogenized by hand in ethanol (95%) for 5 min. The mixture was filtered in the paper filter, and the absorbance was measured from 350 to 800 nm, following the described methodology,^20^ with modifications.

### 3.7 Soil Microbiology

The number of CFU of bacteria and fungi was determined at time zero and at the end of the study (38 days), for the control sample, PS-MPs and BIO-MPs, separately. 1 g portion of soil from each sample (control, Bio-MPs, and PS-MPs) was weighed and dispersed into 9 mL of NaCl solution (0.85 g·mL^–1^), homogenized, and left to rest for sedimentation, and then the supernatant was used for the serial dilution. The diluted samples were individually spread on the Petri dish, incubated at 37 °C for 24 h, and counted. The culture medium for bacteria and fungi counting was nutrient agar and Sabouraud agar, respectively. The tests were carried out in triplicate.

### 3.8 Statistical Analysis

Statistical analysis was assessed by one-way ANOVA or two-way ANOVA followed by Tukey’s post hoc test (*p* < 0.05).

## 4 Results

In this study, the soil was loaded with two MP types to study their influence in *P. vulgaris* L. beans growth, when compared to the soil free of MPs (control soil). The growth of the plants in control soil and in the soil loaded with the MPs was followed, and the data related to the vegetable development such as seedling incidence, leaf, stem and root biomass, stem length, chlorophyll production soil, pH, and number of microorganisms in the soil were measured and compared. The first step of the study was the MP morphology characterization by SEM.

SEM images of MPs revealed a size of about 500 μm for most of the Bio-MPS and PS-MPs particles, as can be seen in Figure 3. After SEM analyses, the MPs were used to grow the beans.

**Figure 3:**
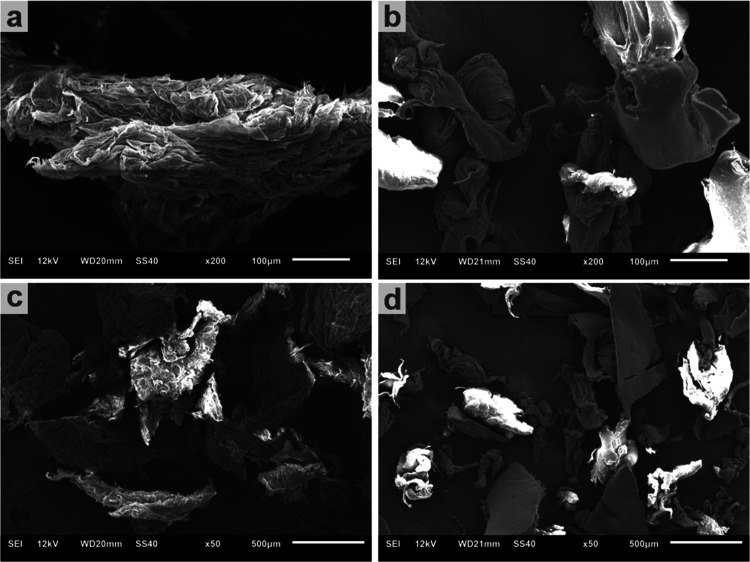
SEM of PS-MPs (a and c) and BIO-MPs (b and d) at two different magnifications, in which the structures are the respective MP.

### 4.1 Seedling Incidence, Leaf, Stem, and Root Biomass

The results of seedling incidence are shown in Figure 4a, as well the pictures of the plants at 3, 5, 7, and 10 days after seedling. On the second day after seedling, about 20% of the seeds emerged when using the soil free of MPs (control soil), 10% emerged in the Bio-MPs soil, and no emergence was found in PS-MPs. At the third day after seedling, 100% of the seeds emerged when using Bio-MPs soil and about 90% for the samples seeded in the PS-MPs soil. About 90% of the samples seeded in control soil emerged at the 10 day time point. The results indicated that both soils loaded with MPS have positively influenced the seeds emergence. One possibility is the aeration and water percolation in the soil with MPS, which could lead the seeds rapidly to emerge. The growth of the plants at this period of time can be visualized in Figure 4b.

**Figure 4:**
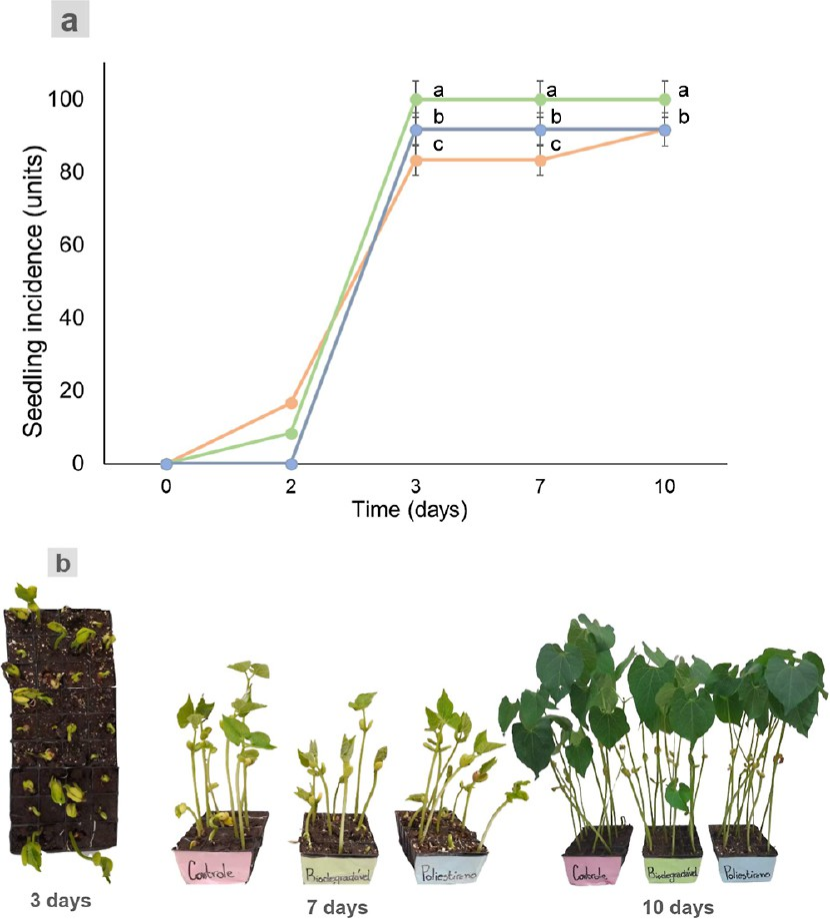
Incidence of the seeds (a) and pictures of the samples at 3, 7, and 10 days time points (b); orange: control, green: BIO-MPs, and blue: PS-MPs. Data represent mean ± standard deviation (*n* = 3). Lines with different letters (a–c) indicate significant differences (*p* < 0.05).

### 4.2 Morphology and Plant Growth after Cultivation

The morphology of the plants and roots of the control, Bio-MP, and PS-MP samples, at 7 (a, c, e) and 38 (b, d, f) days old is shown in Figure 5. The images clearly show the larger leaf and roots size for the control (b) and Bio-MP (d) samples at 38 days, when compared to 7 days samples. Larger leaves and smaller stems were observed for most of the samples (b, d, f), at 38 days, when compared to 7 days samples (a, c, e).

**Figure 5:**
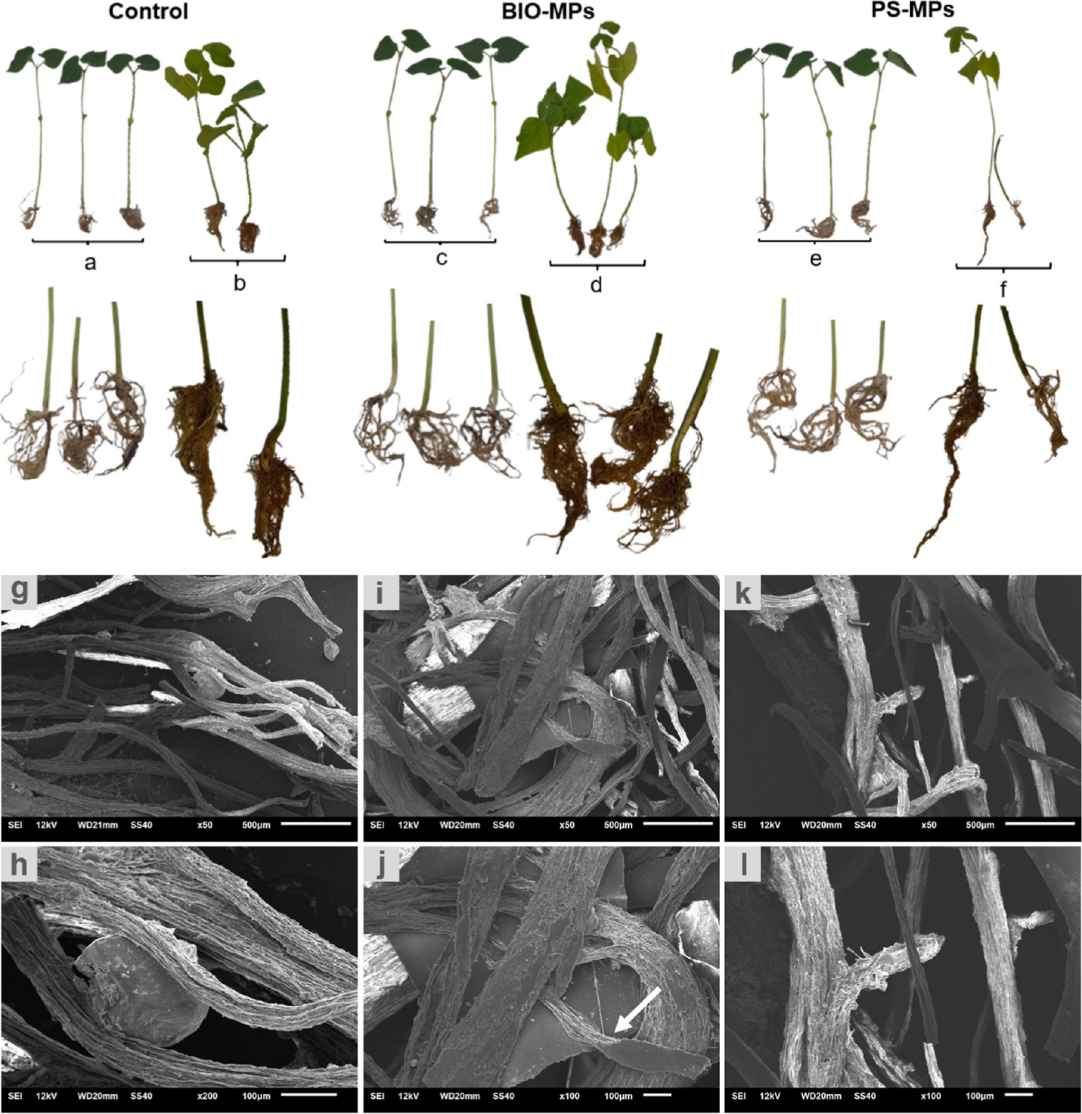
Photographic pictures and SEM microscopy at two magnifications of the roots of the control (a, b, g, and h), BIO-MPs (c, d, i, and j), and PS-MP (e, f, k, and l) samples. Scale bar at g, i, k = 500 μm and at h, j, and l = 100 μm.

The lack of leaves and stems can be seen in PS-MP samples (f) at 38 days. The smaller size of PS-MP root samples at 38 days (f) is remarkable when compared to the other samples at the same period of time.

The morphology of the root was analyzed by SEM in order to try to search for the sites of contact of the microplastic with the root (Figure 5g and l). The control sample (Figure 5g and h) displayed a spherical structure bond to its root, which seems to be the initial state of a rhizobium formation. Bio-MP samples clearly displayed the microplastic entrapped within the root network, as indicated by the arrow (Figure 5i and j). No microplastic was found in the PS-MP sample (Figure 5k and l).

The vegetable growth was evaluated by measuring the leaf, stem, and root biomass and the stem length. The results are shown in Figure 6.

**Figure 6:**
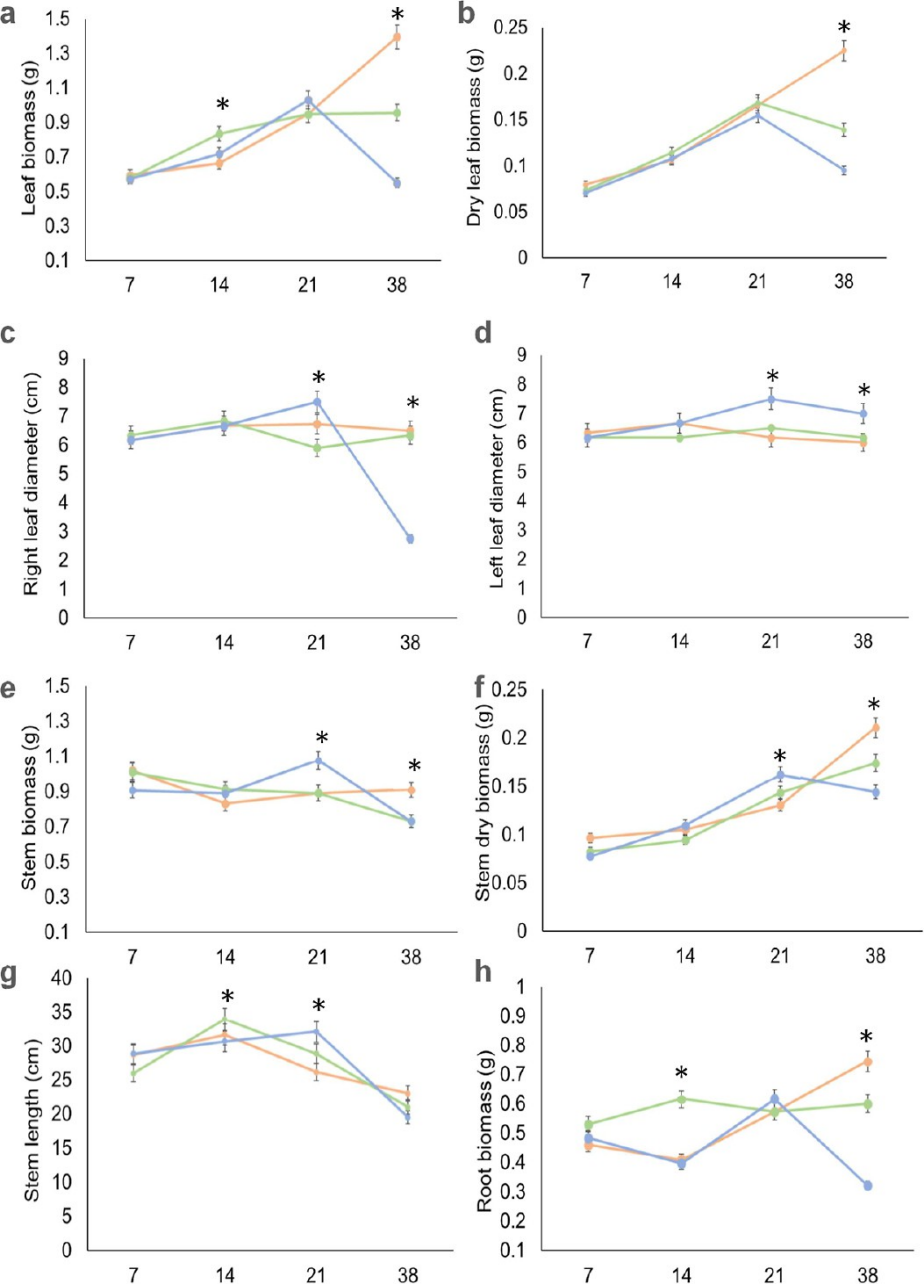
Results of the fresh leaf biomass (a). Dry leaf biomass (b). Length of right leaf (c). Length of left leaf (d). Incidence of stem biomass (e). Dry stem biomass (f). Stem length (g). Root biomass (h). *X* axis corresponds to time (days). Orange: control, green: Bio-MPs, and blue: PS-MPs. Data represent mean ± standard deviation (*n* = 3). Statistical significant differences (*p* < 0.05) are indicated by *.

#### 4.2.1 Leaf Biomass

The results of the fresh and dry leaf biomass measurements are shown in Figure 6a and b, respectively. All the samples displayed a gradual leaf biomass increase from 7 to 21 days time point. The control sample showed a higher biomass at 38 days, followed by Bio-MP and PS-MP samples. PS-MPs, particularly, showed a great decrease in leaf biomass at 38 days. With respect to the PS-MP samples, its second replicate only displayed the stem and root, without leaves and the third replicate did not develop, as shown in Figure 5f.

#### 4.2.2 Leaf Length

The leaf length is shown in Figure 6c (right leaf) and d (left leaf), indicating similar behavior for all the plants until 14 days and some variations at 21 days. Control and Bio-MPs remained stable until 38 days old. PS-MP samples displayed a great decrease in its leaf length, in agreement with the leaf biomass behavior.

#### 4.2.3 Stem Biomass

The results of stem biomass are shown in Figure 6e (fresh) and f (dry). Fresh control samples displayed a constant biomass, in line with time. The dry biomass showed a gradual increase with time for all of the samples; at 38 days, it was higher for the control samples, followed by Bio-MPs and PS-MPs.

#### 4.2.4 Stem Length

The stem grew until the 14th day, as shown in Figure 6g. After that period of time, the control and Bio-MPs showed a decreased growth rate and a decreased length at 21 days. The lower length was evidenced at 38 days for all the samples, in agreement with the images of Figure 5b, d, and f.

#### 4.2.5 Root Biomass

Root biomass, Figure 6h, showed a gradual increase with time for the control sample and remained stable for the Bio-MP samples. PS-MPs showed irregular behavior from 7 to 38 days, with very low biomass at 38 days, in total agreement with the images in Figure 5f.

### 4.3 Chlorophyll and pH Behavior

The absorption spectrum profiles of all the samples at 7 days were similar and displayed predominantly typical features of chlorophyll A, with narrow bands in the blue (∼440 and 470 nm) and red (∼670 and 620 nm) spectral ranges. The bean grown in the PS-MP soil displayed a higher content of chlorophyll, as can be seen by its most intense band, followed by the control and Bio-MPs samples, with lower chlorophyll content, respectively, Figure 7a and b. At 38 days, the spectrum profiles of all the samples were also similar, displaying predominant typical features of chlorophyll A; however, the intensity of the bands was much lower than at 7 days time point. Interestingly, at this time point, the PS-MP sample displayed the lowest intense band, and the BIO-MP sample displayed the highest intense band. Figure 7c shows the pH values of the soil samples over time. From 7 to 21 days, all the samples exhibited pH values within the range considered ideal for growth of the studied plant, ranging from 6.93 to 7.6.^21^ However, on day 38, the samples pH increased, reaching values close to 8, indicating a slight soil alkalinization.

**Figure 7:**
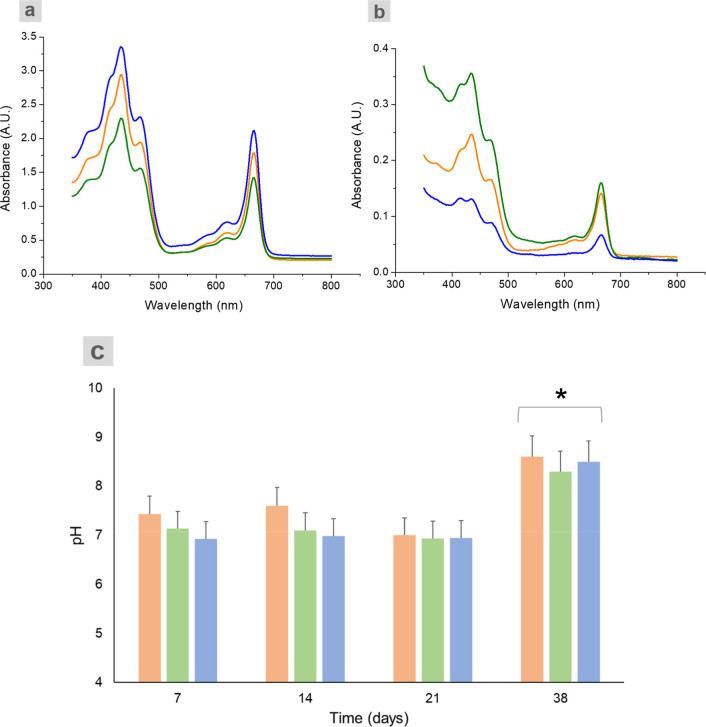
Absorption spectra of the samples leaves ethanolic extract at 7 (a) and 38 (b) days time point and the soil pH measurements at 7, 14, 21, and 38 days (c). The results represent the average of three samples, indicated by the SD bar. Orange: control, green: Bio-MPs, and blue: PS-MPs. Data represent mean ± standard deviation (*n* = 3). Significant differences (*p* < 0.05) are indicated by *.

### 4.4 Microbiological Analysis

The results of the microbiological analyses, showing the number of bacteria and fungi in the soils, can be seen in Figure 8. The number bacteria at zero day time point was about 1.34 × 10^8^ CFU·g^–1^ for the control soil, i.e., the soil free of MPs. After 38 days, all the samples, including the control sample, BIO-MPs and PS-MPS, showed a decreased number of bacteria, at the order of 10^4^ CFU·g^–1^, as shown in Figure 8a. With respect to fungi counting, the control sample showed about 2.0 × 10^4^ CFU·g^–1^ at the beginning of the experiment, i.e., at zero day time point. At 38 days, the number of fungi colonies increased for all of the samples. It was higher for the control sample, with about 1.0 × 10^6^ CFU·g^–1^, followed by Bio-MPs with about 2.0 × 10^5^ CFU·g^–^1 and PS-MPs with about 1.0 × 10^5^ CFU·g^–1^, as shown in Figure 8b. Interestingly, at 38 days, the Bio-MPs and the control samples displayed a higher diversity of fungi, based on their morphologies, when compared to the PS-MP sample soil, as shown in Figure 8c.

**Figure 8:**
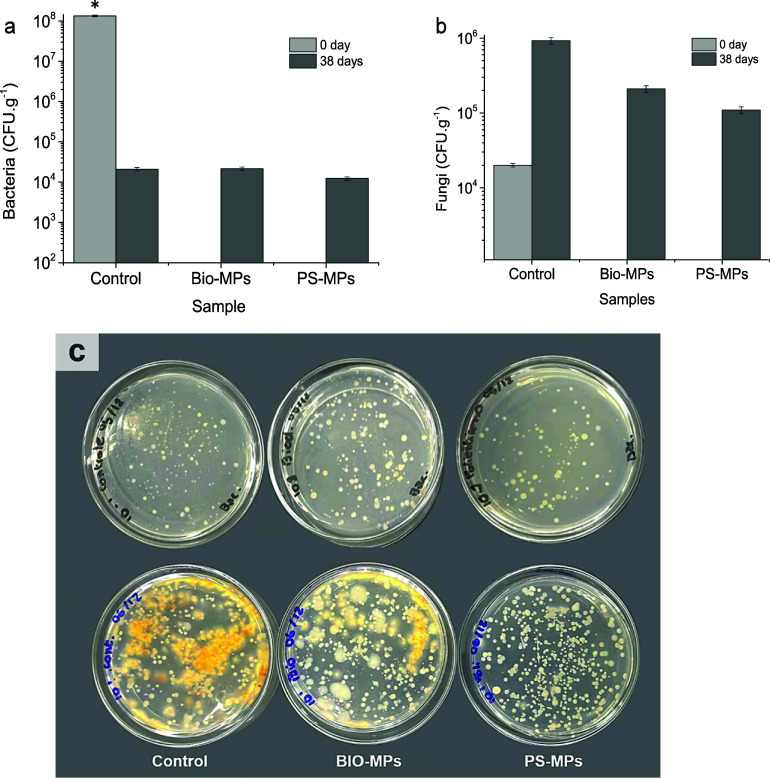
Results of bacteria (a) and fungi (b) counts, and the corresponding photographic images of their respective Petri dishes (100 mm in diameter) showing the grown microorganisms (38 days old). In panel (c), bacteria are shown at the top and fungi at the bottom of the image. Statistical analysis was performed using one-way ANOVA followed by Tukey’s posthoc test; * indicates significant differences between groups (*p* < 0.05).

## 5 Discussion

The seed’s emergence was 100% for Bio-MPs and 90% for the samples in PS-MPs and control soils, until the 10 days time point, as shown in Figure 4. The result is as expected, once most of the seeds have emerged within the expected period of time, with low emergence fail. The morphology of the samples was, in general, influenced by the MPs. Leaf biomass was higher for the control soil samples, followed by Bio-MPs and PS-MPs. At 38 days, only one PS-MP sample displayed leaves, suggesting the negative influence of PS-MPs on their development. These data indicated that the soil free of MP was capable of generating more leaf biomass than the plant grown in the soil loaded with MP for the studied period of time.

The measurement of leaf length showed similar values and did not change among the samples for 21 days. The dry stem biomass increased gradually for all samples until day 21, and the samples from the control and Bio-MP soils followed this behavior until 38 days. At this period of time, the samples from the control soil displayed the highest biomass. The sample from PS-MP soil displayed a marked decrease in biomass at 38 days, in agreement with leaf biomass.

Stem biomass, stem length, and root biomass followed similar behavior, in which the samples grown on the control soil and Bio-MP soil reached similar values when compared to each other along the time. However, PS-MPs displayed a distinct pattern, with much lower values for all the measured parameters at 38 days, indicating their harmful influence on the development of the plant samples. In general, the results of the morphological analyses evidenced the negative influence of the soil loaded with PS-MPs on the development of *P. vulgaris*, which led mainly to failure on the development of leaves and stems and decreased the root development when compared to the samples grown on control or Bio-MPs soils. The negative influence of PS-MPs in the dry root mass of *Vigna radiata* L. (mung bean) was already described in the literature, however, in that study, it was associated with Pb effects.^22^ According with the literature, PS-MPs can also display inhibitory effects on the vegetable development, in the germination and seedling growth phases.^23^ Further issues are also described. For instance, polypropylene and rubber crumb MPs were capable of inhibiting N absorption in peanut plants in just 10 days. These MPs damaged the plasma membranes of root cells, causing oxidative stress and decreasing the number of xylem vessels, leading to the inhibition of N absorption by the roots.^24^ High-dose of PE MPs suppressed the N fixation potential of soil bacteria.^25^ PET and LDPE MPs reduced the abundance and diversity of the soil bacterial community after 42 days. The soil that received 3% LDPE MPs treatment showed an approximately 8-fold increase in soil respiration compared to other treatments and the control soil.^26^ High doses of polybutylene adipate terephthalate (PBAT-BD) increased the alpha diversity indices of bacterial and fungal communities compared to the control in just over 11 weeks. Soil respiration increased as the concentration of this MP increased.^27^

The soil pH remained in the range of 6.93 to 7.6 for all the samples, from days 0–21, which is suitable for growing beans because in such pH range (6–7), the nutrients are available. At 38 days time point, the pH of all soil samples was slightly alkaline, higher than 8. In this pH range, the availability of macro and micronutrients, such as N, P, S, B, Zn, Cu, and Mn is lower, leading to its decreased absorption.^21^ The deficiency of these nutrients influences vegetable health, and the change in leaves color is clear evidence of the problem, as evidenced by the yellowish-green color of the bean leaves in Figure 5b, d, and f. This result was strictly related to the chlorophyll behavior profile shown in Figure 7. At the beginning of the experiment, all the samples displayed intense bands attributed to chlorophyll, and at 38 days, all of them were strongly decreased, in total agreement with the results of the pH measurements. In this study, the PS-MP sample displayed the highest chlorophyll bands at 7 days and lowest at 38 days when compared to Bio-MPs and control samples. The chlorophyll production in vegetables is associated with their functional photosynthetic systems and is strictly associated with the greenness of the vegetables, which level depends on the light exposition or on the vegetable health. Leaf greenness is an indicator of senescence, stress, and damage to the plant and the photosynthetic system. Leaves become more yellowish—green as the senescence progresses.^28^ The results of the experiments suggest its fast development at the beginning of the experiment and also its fast senescence. The literature describes that PS-MPs associated with Pb decreased the chlorophyll content of *V. radiata* L.^22^ and also reduced the chlorophyll content of wheat by 15%.^23^

The number of microorganisms (fungi and bacteria) in the soil loaded with the MP clearly changed from the beginning to the 38 day time point. The number of bacteria decreased for all of the samples, including the control, at the end of the experiment. This result indicated that the MPs did not influence the number of bacteria in the soil once the behavior of the control soil was quite similar to the behavior of the MP-treated soils. The number of fungi colonies increased for all the samples, including the control, after 38 days of cultivation, when compared to time zero, being higher for the control, followed by Bio-MPs and PS-MPs. This result clearly indicated the negative influence of the MPs on fungi number in the samples contaminated with it, once the fungi number was about ten times lower than the number found in the control sample. In addition to the decreased number of fungi in soil contaminated with MPs, the diversity of the colonies morphologies was also evidently lower for the soils contaminated with MPs, and much lower for PS-MPs, as shown in Figure 8c. The literature shows that the soil treatment with PS significantly downregulated the expression of phenylalanine ammonia-lyase, *trans*-cinnamate 4-monooxygenase, hydroxycinnamoyl transferase, cinnamoyl-CoA reductase, peroxidase, and other enzyme-related genes.^29^ The study suggests that PS decreased the stability of the cell wall and cell membrane by lowering the rate of lignin synthesis and increasing the relative permeability of the plasma membrane.^29^ Also, there is evidence that LDPE-MPs and Bio-MPs exert deep effects on the rhizosphere bacterial communities, and these effects can be associated with the effects of soil nutrient cycling and plant health in agroecosystems. Bio-MPs and LDPE-MPs showed significantly higher α-diversity microorganisms than the control, affecting relative abundance at the family level, i.e., compared to the control.^30^

In general, the main limitation of this work was the decreased development rate of the vegetables after 21 days, which maybe could be avoided if it was planned to supplement the soil with fertilizers. However, we did not supplement it in an attempt to avoid its influence on the results. Another possible improvement would be to grow the vegetables for a longer period of time aiming to determine the flowering and beans production; however, as it would be a long experiment, it must be performed in a larger area and with a higher number of samples. Further investigations at the molecular level, in particular, transcriptomic or proteomic analyses, could help identify the genes and metabolic pathways involved in stress response, nutrient uptake, and photosynthetic regulation under microplastic exposure. Such omics-based approaches could clarify how PS-MPs influence plant physiology at the cellular and molecular scales and reveal potential biomarkers of soil or plant health associated with microplastic contamination. The microbial diversity can also be studied by transcriptomics, increasing the consistency of the data.

## 6 Conclusion

The results of this study showed that soils contaminated with polystyrene MP (PS-MPs) negatively affect the early growth of *P. vulgaris*, particularly reducing leaf and stem biomass, altering root formation, and accelerating senescence. In contrast, soils loaded with biodegradable MP (Bio-MPs) showed less detrimental effects, exhibiting plant responses that were more comparable to those of the control soil. The observed changes in the chlorophyll content, pH stability, and microbial dynamics further indicate that PS-MPs can disrupt nutrient availability and microbial balance, leading to impaired plant health and altered soil function. These findings contribute to understanding how persistent plastic contaminants influence agroecosystems and food production. The clear contrast between PS-MPs and Bio-MPs highlights the importance of developing and adopting environmentally safer materials. Such evidence may support the design of strategies and regulations to mitigate microplastic pollution in agricultural soils. Moreover, this research reinforces the need to integrate microplastic monitoring into soil quality assessment frameworks and to promote sustainable agricultural practices that safeguard soil health and biodiversity. Future studies should focus on longer cultivation periods and evaluate crop productivity and soil fertility under controlled and field conditions, providing a stronger basis for decisions and sustainable land management strategies.

## Data Availability

The data will be made available on request.
